# The relationship between preoperative serum indirect bilirubin and postoperative delirium in geriatric patients undergoing joint replacement

**DOI:** 10.1371/journal.pone.0320719

**Published:** 2025-03-27

**Authors:** Rili Yu, Mi Yang, Juan Chen, Fan Zhang

**Affiliations:** 1 Department of Anesthesiology, The Third Xiangya Hospital, Central South University, Changsha, Hunan, People’s Republic of China; 2 Department of Endocrinology, The First Hospital of Changsha, Changsha, Hunan, People’s Republic of China; Kwame Nkrumah University of Science and Technology Faculty of Pharmacy and Pharmaceutical Sciences, GHANA

## Abstract

**Background:**

Postoperative delirium (POD) is one of the most common complications in geriatric patients following surgery. Physiological concentration of bilirubin possesses anti-inflammatory, antioxidant and neuroprotective effects, which are important protective mechanisms against POD. This study aimed to explore the relationship between preoperative serum bilirubin and POD in geriatric patients undergoing joint replacement.

**Methods:**

Geriatric patients who underwent hip or knee joint replacement surgery under intrathecal anesthesia were included. These patients had American Society of Anesthesiologists (ASA) grades I to III. The patients with a history of psychiatric or neurological disorders, infectious diseases or sepsis, hemolytic anemia, liver diseases, performed general anesthesia or intrathecal anesthesia combined with general anesthesia, or insufficient surgical information were excluded. Patients’ age, gender, weight, height, ASA classification, liver function and bilirubin within one week before surgery, preoperative Mini–Mental State Examination (MMSE) scores, surgical type, dosage of medications, intraoperative bleeding volume, postoperative average numeric rating scale (NRS) pain scores, the occurrence of POD and Delirium Rating Scale–Revised–98 (DRS–R–98) scores for POD were collected.

**Results:**

A total of 269 patients were eligible for inclusion in the study, with 23.05% (62/207) exhibiting POD. Patients with POD exhibited higher age and ASA classification, and had lower weight, serum total serum bilirubin (TBIL) and indirect bilirubin (IBIL) within one week before surgery, and preoperative MMSE scores (all *p* < 0.05). Univariate logistic regression analysis showed that the above variables were correlated with the occurrence of POD (all *p* < 0.05). Multivariate logistic regression analysis revealed that age was a risk factor (*p* < 0.001, OR = 1.14, 95% CI [1.07*–*1.21]), while weight (*p* = 0.041, OR = 0.96, 95% CI [0.92*–*0.99]), IBIL levels within one week before surgery (*p* = 0.012, OR = 0.65, 95% CI [0.47–0.91]) and preoperative MMSE scores (*p* < 0.001, OR = 0.84, 95% CI [0.78*–*0.91]) served as protective factors against the occurrence of POD. The serum IBIL concentration within one week before surgery was performed receiver operating characteristic (ROC) curve analysis. The estimated cutoff value for predicting the occurrence of POD was 6.65 μmol/L, and area under the curve (AUC) was 0.63. Patients with preoperative serum IBIL concentration below 6.65 μmol/L had a higher incidence of POD.

**Conclusion:**

Patients with lower preoperative serum IBIL levels exhibited a higher incidence of POD in geriatric patients undergoing joint replacement. Low serum IBIL was a risk factor and a predictor for the occurrence of POD.

## Introduction

Postoperative delirium (POD) represents one of the most prevalent complications among geriatric patients following surgery, with an incidence rate as high as 24% [1]. It is characterized by acute and fluctuating impairment of attention and consciousness [2]. POD has been linked to extended hospital stays, escalated healthcare expenses, diminished quality of life and self–care capabilities, and an increased risk of developing postoperative dementia and mortality [3]. Due to its clinical significance, a series of interventions have been implemented to mitigate intra– and postoperative risk factors associated with POD, including benzodiazepines, anticholinergic medications, intraoperative bleeding, postoperative pain, and perioperative infection, etc [4]. However, the effectiveness of these interventions is inconsistent, as some geriatric patients, undergoing identical procedures and anesthetics, still experience POD. It suggests that preoperative risk factors for POD, particularly the decline in brain reserve of patients, may be pivotal in the onset and progression of POD [5]. To date, no accessible predictors for POD have been proposed. Therefore, it is crucial to find novel predictors to prevent and reduce the occurrence of POD.

Bilirubin is the terminal product of heme metabolism. Serum indirect bilirubin (IBIL), is commonly referred to as unconjugated bilirubin, primarily originates from the breakdown of red blood cells and is transformed into direct bilirubin (DBIL) through binding to glucuronic acid in the liver. Previous researches have suggested that excessive elevated serum bilirubin induces brain damage and has a negative impact on neurodevelopment [6,7]. However, an increasing number of studies have demonstrated that bilirubin exhibits potent anti-inflammatory, antioxidant, and neuroprotective effects [8–11]. In healthy individuals, lower serum bilirubin levels are associated with an increased risk of white matter lesions in the brain [12]. In males with schizophrenia, the lower the total serum bilirubin (TBIL) levels, the more severe the cognitive impairment [13]. Vitro study has confirmed that bilirubin can prevent dopaminergic neuron death by acting on tumor necrosis factor alpha (TNF–α) [14]. Nevertheless, whether preoperative bilirubin levels are associated with POD remains unclear. Therefore, we investigated the relationship between preoperative serum bilirubin levels and POD in geriatric patients undergoing joint replacement.

## Methods

### Study design and participants

**Fig 1:**
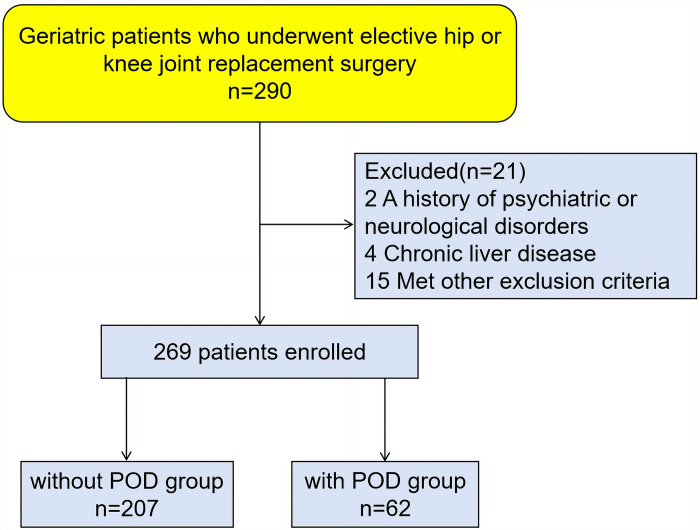
Flow chart for patient selection.

This was a retrospective observational study. Geriatric patients who underwent elective hip or knee joint replacement surgery at the Third Xiangya Hospital of Central South University between April 2022 and January 2024, were included in this study. Patients were eligible for inclusion in the study if they satisfied all of the following criteria: (1) sixty–five years old or older; (2) American Society of Anesthesiologists (ASA) grade I to III; (3) intrathecal anesthesia; (4) effectively communicate with researchers and complete all relevant scales. The exclusion criteria for the study were as follows: (1) with a history of psychiatric or neurological disorders; (2) baseline conditions of infectious diseases, or sepsis; (3) hemolytic anemia; (4) chronic liver disease, cirrhosis, or liver tumors; (5) alanine aminotransferase (ALT) or aspartate aminotransferase (AST) levels more than two times of the upper limit of the normal range; (6) patients who performed general anesthesia or intrathecal anesthesia combined with general anesthesia; (7) insufficient surgical information, lack of data on IBIL, DBIL, and TBIL. In total, 269 patients were included in our study. The patient selection process was illustrated in a flow chart (Fig 1).

This study was approved by the Institutional Review Board of the Third Xiangya Hospital of Central South University (Date: 01/08/2024, Decision No: K24662) and was carried out in accordance with the Declaration of Helsinki. Due to the retrospective nature of this study, the ethics committee approved a waiver for the informed consent requirement concerning the utilization of existing data, in compliance with both national legislation and institutional guidelines. For the purpose of privacy protection in this study, personal identification data of the enrolled participants was anonymized and substituted with a coding system.

### Data collection

The date for accessing data for research purposes is 05/08/2024. A trained researcher was responsible for collecting preoperative bilirubin levels and other relevant data, which were then entered into the database. Another professional anesthesiologist documented the POD status of patients. The two researchers mentioned above were unaware of each other’s data and results. Data monitoring and source-data verification were conducted by a third researcher according to a predefined plan.

The following clinical data were retrospectively reviewed: age, gender, weight, height, past medical history, ASA classification, preoperative cognitive function, liver function and bilirubin data within one week before surgery. In addition, we collected data on the type of surgery performed, dosage of medications, intraoperative bleeding volume, the average NRS pain scores of patients within three days after surgery, the occurrence and severity of POD in the first five days after surgery.

### Anesthesia methods

Anesthesia was managed by specialized anesthesiologists. All patients underwent elective hip or knee joint replacement surgery under intrathecal anesthesia with or without nerve block. For postoperative analgesia, patients were typically fitted with electronic patient-controlled analgesia (PCA) pumps delivering sufentanil.

### Preoperative cognitive function evaluation

Preoperative cognitive function was measured by Mini-Mental State Examination (MMSE) scores, which was a brief screening tool commonly used to assess cognitive impairment and to detect dementia. Comprising a series of questions and tasks, it evaluated various cognitive domains, including orientation, memory, attention and calculation, recall, and language abilities. The total score range for MMSE was 0 to 30 points. Scores ranging from 0 to 9 were typically indicative of severe cognitive impairment, those from 10 to 20 may suggest moderate cognitive impairment, while scores of 21 to 30 were generally regarded as normal cognitive function or only mild impairment.

### POD evaluation

The occurrence of POD was assessed utilizing the Confusion Assessment Method (CAM), which included four clinical criteria: (1) acute onset and fluctuating course, (2) inattention, (3) disorganized thinking, and (4) altered level of consciousness. To define the occurrence of delirium, (1) and (2) must both be satisfied in addition to (3) and/or (4). Patients were classified as POD if they had a positive CAM on any day throughout the first five postoperative days.

The severity of the POD was determined using the Delirium Rating Scale–Revised-98 (DRS-R-98). This scale comprised 13 indicators, each of which was classified into various levels based on the severity of symptoms, with the cumulative score representing the severity of symptoms. The higher the cumulative score, the more severe the delirium. CAM and DRS-R-98 were investigated by clinical researchers who had undergone comprehensive training and were able to accurately apply these assessment tools. The evaluation outcomes were meticulously reviewed and professionally interpreted by a psychiatrist.

### Postoperative pain management and evaluation

Acute postoperative pain was managed and evaluated by a professional acute pain services (APS) team. They implemented follow-up assessments at 4h, 24h, 48h, and 72h after surgery. Between these intervals, the APS staff monitored the usage of PCA pumps through the center to gauge the patient’s pain condition. If the press frequency of PCA was increased abnormally, the APS staff would go to the ward to assess the patient’s pain situation and handle it, maintaining the NRS score of less than 3 points. The NRS consisted of eleven evenly spaced numbers from 0 to 10, with 0 representing “pain–free” and 10 representing “the most intense pain”. The higher the number, the more severe the pain. We analyzed the average NRS pain scores of patients across various timepoints within three days after surgery.

### Statistical analysis

The Kolmogorov–Smirnov test was employed to ascertain whether the continuous variables followed a normal distribution. Data were expressed as means ±  standard deviations for continuous variables with normal distribution, medians and interquartile ranges for non–normally distributed continuous variables, and frequencies for enumeration data. The unpaired t test or the Mann–Whitney U test was respectively performed to evaluate the differences between two groups of continuous variables with normal or non–normal distribution. Enumeration data were compared with the χ^2^ test. Univariate logistic regression analysis was employed to examine the relationship between each variable and the occurrence of POD. Then multivariate logistic regression analysis was performed to identify independent biomarkers of POD. Concurrently, corresponding odds ratios (ORs) and 95% confidence intervals (CIs) were computed. Receiver operating characteristic (ROC) curve was drawn for the quantitative variable significantly associated with the occurrence of POD, and optimal decision threshold was determined. *P* < 0.05 was considered statistically significant. Statistical analyses were performed using SPSS 25.0 (SPSS Inc., Chicago, IL, USA) with copyright license.

## Results

A total of 269 patients over the age of sixty-five who underwent joint replacement were included in this study. 127 patients underwent hip replacement and 142 patients underwent knee replacement. Among them, 62 (23.05%) experienced an incidence of POD. All patients received intrathecal anesthesia without the use of benzodiazepines or dexmedetomidine for sedation, and no postoperative wound infections occurred. Under the care of our APS team, all patients maintained pain-free or mild pain status within seventy–two hours after surgery*.*

### Demographic and clinical characteristics

Table 1 presents a comparative analysis of demographic and clinical data between patients with and without POD. Patients with POD exhibited higher age, ASA classification, and postoperative DRS–R–98 scores than those without POD (*p* < 0.001). Furthermore, patients with POD had lower body weight, serum TBIL and IBIL levels within one week before surgery, and preoperative MMSE scores compared to those without POD (*p* < 0.05). There were no significant differences between the two groups in other variables.

**Table 1:** Demographic and clinical characteristics of patients with and without POD.

Variables	Without POD	With POD	t/ χ^2^	*p*-value
Patients, n	207	62		
Age (years)	71.03 ± 5.35	76.65 ± 8.03	6.40	<0.001^#^
Male/ female, n	51/ 156	18/ 44	0.48	0.487
Weight (kg)	60.28 ± 10.90	54.23 ± 7.97	4.06	<0.001^#^
Height (cm)	157.40 ± 6.08	156.90 ± 7.83	0.55	0.585
ASA classification				
I/ II/ III/ IV, n	0/ 90/ 117/ 0	0/ 12/ 50/ 0	11.79	<0.001^#^
ALT within one week before surgery (U/L)	16.68 ± 7.46	15.89 ± 3.33	0.81	0.417
AST within one week before surgery (U/L)	22.51 ± 8.96	21.56 ± 7.26	0.76	0.447
TBIL within one week before surgery (μmol/L)	12.96 ± 7.63	10.82 ± 5.53	2.05	0.042 *
DBIL within one week before surgery (μmol/L)	4.33 ± 3.33	3.87 ± 2.15	1.01	0.312
IBIL within one week before surgery (μmol/L)	8.63 ± 4.70	6.95 ± 3.63	2.59	0.010 *
Preoperative MMSE scores	25.29 ± 4.09	21.63 ± 5.57	5.66	<0.001^#^
Type of surgery				
Hip/ knee replacement, n	93/ 114	34/ 28	1.88	0.170
Perioperative use of atropine, n (%)	13 (6.28%)	5 (8.06%)	0.24	0.622
Intraoperative bleeding volume (ml)	148.30 ± 62.50	144.50 ± 61.13	0.42	0.678
NRS pain scores within three days after surgery	1.50 ± 0.90	1.45 ± 0.90	0.35	0.724
Postoperative DRS–R–98 scores	8.16 ± 2.99	21.66 ± 4.84	26.64	<0.001^#^

Data are expressed as mean ± standard deviation or number (%). POD, postoperative delirium; ASA, American Society of Anesthesiologists; ALT, alanine aminotransferase; AST, aspartate aminotransferase; TBIL, total bilirubin; DBIL, direct bilirubin; IBIL, indirect bilirubin; MMSE, mini*–*mental state examination; NRS, numeric rating scale; DRS-R-98, delirium rating scale*–*revised*–*98. *  *p* <  0.05, # *p* < 0.001.

### Binary logistic regression analysis of factors related to POD

Univariate logistic regression analysis showed that age (*p* < 0.001, OR: 1.14, 95% CI: 1.09*–*1.19), weight (*p* < 0.001, OR: 0.94, 95% CI: 0.91*–*0.97), ASA classification (*p* = 0.001, OR: 3.21, 95% CI: 1.61*–*6.37), TBIL (*p* = 0.046, OR: 0.95, 95% CI: 0.90–0.99) and IBIL (*p* = 0.013, OR: 0.90, 95% CI: 0.83*–*0.98) within one week before surgery, and preoperative MMSE scores (*p* < 0.001, OR: 0.86, 95% CI: 0.81*–*0.91) were related to the occurrence of POD in patients underwent joint replacement (Table 2). Multivariate logistic regression analysis revealed that age (*p* < 0.001, OR: 1.14, 95% CI: 1.07*–*1.21) was a risk factor, while weight (*p* = 0.041, OR: 0.96, 95% CI: 0.92*–*0.99), IBIL levels within one week before surgery (*p* = 0.012, OR: 0.65, 95% CI: 0.47*–*0.91) and preoperative MMSE scores (*p* < 0.001, OR: 0.84, 95% CI: 0.78*–*0.91) served as protective factors against the occurrence of POD in patients underwent joint replacement (Table 2).

**Table 2:** Binary logistic regression analysis results of factors related to the occurrence of POD.

Variables	Univariate Analysis			Multivariate Analysis		
	*p*-value	Unadjusted OR	95% CI	*p*-value	Adjusted OR	95% CI
Age (years)	<0.001^#^	1.14	1.09–1.19	<0.001^#^	1.14	1.07–1.21
Weight (kg)	<0.001^#^	0.94	0.91–0.97	0.041 *	0.96	0.92–0.99
ASA classification, n	0.001^#^	3.21	1.61–6.37	0.176	0.57	0.25–1.29
TBIL within one week before surgery (μmol/L)	0.046 *	0.95	0.90–0.99	0.157	1.15	0.95–1.40
IBIL within one week before surgery (μmol/L)	0.013 *	0.90	0.83–0.98	0.012 *	0.65	0.47–0.91
Preoperative MMSE scores	<0.001^#^	0.86	0.81–0.91	<0.001^#^	0.84	0.78–0.91

POD, postoperative delirium; ASA, American Society of Anesthesiologists; TBIL, total bilirubin; IBIL, indirect bilirubin; MMSE, mini–mental state examination. *  *p* < 0.05, # *p* < 0.001.

### Preoperative serum IBIL was a novel predictor for the occurrence of POD in geriatric patients undergoing joint replacement

**Fig 2:**
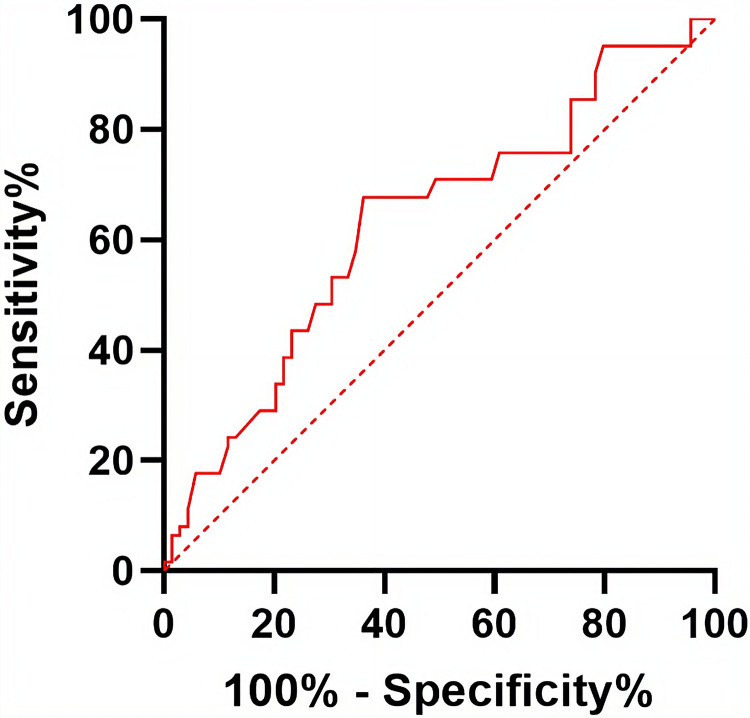
ROC curves for predicting the occurrence of POD using serum IBIL concentration measurements taken within one week prior to surgery.

Since serum TBIL is the sum of serum IBIL and DBIL, TBIL is inherently influenced by IBIL. To determine the cutoff value for predicting the occurrence of POD, ROC curve analysis was conducted on the serum IBIL concentration within one week before surgery. The estimated cutoff value for predicting the occurrence of POD was 6.65 μmol/L (sensitivity: 0.68; specificity: 0.64; area under the curve (AUC) = 0.63 (0.55 to 0.71)) (Fig 2). To assess whether this cutoff value was significantly associated with the incidence of POD, serum IBIL concentration within one week before surgery were divided into two groups based on the cutoff value: group one with 2.40–6.60 µmol/L and group two with 6.70–23.90 µmol/L. The incidence of POD in group two was significantly lower than that in group one, with rates of 13.16% and 35.90% respectively (*p* < 0.001) (Table 3). The cutoff value of serum IBIL concentration within one week before surgery could predict the occurrence of POD.

**Table 3:** Comparison of POD incidence between different levels of serum IBIL.

IBIL (μmol/L)	Patients, n	without POD	with POD	POD incidence (%)
2.40–6.60	117	75	42	35.90
6.70–23.90	152	132	20	13.16

POD, postoperative delirium; IBIL indirect bilirubin.

## Discussion

This study was the first time to evaluate the relationship between preoperative bilirubin levels and POD. In our study, the incidence of POD was 23.05% in geriatric patients after joint replacement surgery, aligning with previous researches [1,15]. Notably, we discovered that serum TBIL and IBIL levels within one week prior to surgery were correlated with the occurrence of POD. Low serum IBIL was a risk factor for the occurrence of POD. Additionally, Age emerged as a risk factor for POD, and body weight and preoperative MMSE scores acted as protective factors against POD in geriatric patients after joint replacement.

POD is one of the most common postoperative complications in geriatric patients, with a complex pathogenesis. Neuroinflammation and oxidative stress are currently recognized as pivotal mechanisms of POD [16–19]. The research has revealed that elevated levels of preoperative inflammatory markers, including the neutrophil-to-lymphocyte ratio and interleukin-6, are positively correlated with the development of POD [20, 21]. Postoperative CRP level is an independent predictive indicator for POD among elderly colorectal cancer patients [18]. Notably, bilirubin at its physiological concentration, has been shown to possess potent anti-inflammatory and neuroprotective properties in recent years [10,22]. Bilirubin suppresses the activation and functions of T-cells, and inhibits the expression of pro-inflammatory cytokines [14,23]. Particularly, IBIL, a lipophilic compound with a small molecular weight, easily crosses the blood-brain barrier to exert neuroprotective effects [24]. Furthermore, bilirubin plays an important role in the endogenous antioxidant system [25]. It effectively protects neurons from free radical damage [10]. Based on the anti-inflammatory and antioxidant properties of bilirubin, an increasing number of studies have established a correlation between serum bilirubin levels and a range of neurological and psychological disease, including white matter lesions in the brain [12], cognitive function in individuals with schizophrenia [13], and depression [26]. Our investigation revealed that low serum IBIL within one week before surgery was a risk factor for POD, which may be associated with reduced anti-neuroinflammatory and antioxidant capabilities. Preoperative low serum IBIL may serve as a predictor for identifying individuals who are at risk of experiencing POD.

Our findings suggested that older age, weight loss, and preoperative cognitive impairment were susceptible to POD in geriatric patients after joint replacement, consistent with previous reports [27,28]. This could be attributed to the decline in brain reserve, increase in brain fragility, and reduction in tolerance to surgery and anesthesia in these patients [28,29].

It is important to note that this study has certain limitations. Firstly, the selection criteria were limited to geriatric patients undergoing joint replacement. It may be necessary to verify this correlation and explore its possible mechanisms in patients of varying ages and other surgical procedures. Secondly, although CAM and DRS-R-98 are widely recognized and used in clinical practice for assessing delirium, the training and expertise of evaluators may affect the consistency and reliability of the assessment. Finally, this study was a single-center, retrospective analysis. Larger sample sizes are required to further confirm our findings through prospective and multicenter studies.

## Conclusion

Patients with lower preoperative serum IBIL levels exhibited a higher incidence of POD in geriatric patients undergoing joint replacement. Low serum IBIL served as a risk factor and a predictor for POD.
